# Circular *DDX10* is associated with ovarian function and assisted reproductive technology outcomes through modulating the proliferation and steroidogenesis of granulosa cells

**DOI:** 10.18632/aging.202699

**Published:** 2021-03-19

**Authors:** Hongcai Cai, Tianli Chang, Yamin Li, Yinzhao Jia, Huiying Li, Mengdi Zhang, Ping Su, Ling Zhang, Wenpei Xiang

**Affiliations:** 1 Institute of Reproductive Health/Center of Reproductive Medicine, Tongji Medical College, Huazhong University of Science and Technology, Wuhan 430030, Hubei, China; 2 Department of Urology, The First Affiliated Hospital of Sun Yat-Sen University, Guangzhou 510080, Guangdong, China; 3 Department of General Surgery, Union Hospital, Tongji Medical College, Huazhong University of Science and Technology, Wuhan 430030, Hubei, China

**Keywords:** circDDX10, ovary, granulosa cell, assisted reproductive technology, aging

## Abstract

circRNAs are present in human ovarian tissue, but how they regulate ovarian function remains unknown. In the current study, we investigated the levels of circRNAs in granulosa cells (GCs) derived from human follicular fluid, explored their correlation with female ovarian reserve function and clinical outcomes of assisted reproduction technique (ART), and investigated their effects on the biological functions of GC cell lines (COV434) *in vitro*. We identified that the levels of *circDDX10* in GCs decreased gradually with aging (*P* < 0.01) and was positively correlated with AMH (r = 0.45, *P* < 0.01) and AFC (r = 0.32, *P* < 0.01), but not with FSH and estradiol (*P* > 0.05). Additionally, *circDDX10* was related to the number of oocytes obtained, and good quality embryo rates. Silencing *circDDX10* in GCs could markedly up-regulate the expression of apoptosis-related factors, reduce cell proliferation activity, inhibit the expression of steroid hormone synthesis-related factors, and prohibit the synthesis of estradiol. On the contrary, over-expression of *circDDX10* had the opposite effect. *circDDX10* is expected to become a novel biomarker for predicting the outcomes of ART, and may participate in the regulation of ovarian function by affecting the proliferation and apoptosis of GCs and steroid hormone synthesis.

## INTRODUCTION

Ovarian senescence, characterized by the reduction of the quantity and quality of oocytes, is the key factor affecting the outcomes of assisted reproductive technology (ART) [1]. Granulosa cells (GCs), as an important component of follicles, provide a basic follicular microenvironment that affects oocyte development [2]. Apoptosis of GCs is closely related to follicular atresia, which is crucial to follicle selection [3]. Moreover, various active peptides secreted by GCs, such as inhibin, activin, follistatin, *etc.*, can also regulate follicle development [4]. Many studies have tried to use the transcriptome of GCs as a non-invasive method to deeply understand the microenvironment around oocytes, as well as predict the developmental potential of oocytes [5, 6]. However, due to species heterogeneity and the instability of linear RNAs, there is a lack of consistency between studies.

Non-coding RNAs (ncRNAs), including miRNA, piRNA, and lncRNA, are key members of the epigenetic regulation network [7, 8]. Recently, researchers have discovered their involvement in the regulation of ovarian function [9, 10]. More importantly, a new type of ncRNA, circular RNA (circRNA), has been identified in the ovary of various mammals. This may relate to ovarian activation and oviposition [11–13]. circRNAs have already been proven as a new kind of biomarker in various human diseases due to their particular biological properties. For example, circRNAs are resistant to RNase R digestion as compared to their linear transcripts [14]. Interestingly, one study recently reported circRNA profiles in human GCs during maternal aging and tried to uncover age-related circRNA variations that potentially reflect decreased oocyte competence [15]. They revealed that age-related up-regulation of circRNA_103827 and circRNA_104816 might be potential indicators of a compromised follicular micro-environment. This could be used to predict IVF prognosis, although the mechanism remains to be explored [15]. Another study demonstrated that circINHA promoted GCs proliferation and inhibited GCs apoptosis via connective tissue growth factor as a competing endogenous RNA that directly bound to miR-10a-5p [16]. More recently, researchers found that loss of hsa_circ_0118530 inhibited KGN cell (human granulosa-like tumor cell line) injury by sponging miR-136 [17]. In our previous work, we explored the potential roles of circRNAs in human ovarian senescence using human ovarian samples [18]. We found the ovary-derived circRNA, *circDDX10*, was highly expressed in human GCs, and that it gradually decreased with aging. Bioinformatics demonstrated that *circDDX10* could act as miRNA sponges to participate in the process of ovarian aging.

The aim of this study was to further elucidate the role of ovary-derived *circDDX10* on the biological behaviours of GCs that could potentially reflect oocyte competence, thus offering novel insights into the complex molecular network of ovarian senescence.

## RESULTS

### Baseline information of the participants

We collected 239 human follicular fluid samples. After extracting GCs by method of Percoll density gradient centrifugation, a total of 210 women were enrolled in this study. The participants’ demographics and baseline characteristics, including age, BMI, duration of infertility, female baseline hormone level, ovarian stimulation protocol, and treatment method are presented in Table 1.

**Table 1:** Demographic and clinical characteristics of the enrolled participants (n = 210).

**Variable**	**Mean**	**SD**	**n (%)**
Age, mean	31.7	4.7	
20–25			17 (8.1)
26–30			75 (35.7)
31–35			73 (34.8)
36–40			37 (17.6)
> 40			8 (3.8)
BMI ^a^	22.3	3.6	
< 18.5			24 (11.5)
18.5–24.9			142 (68.3)
25-27.9			26 (12.5)
> 28			16 (7.7)
Duration of infertility (years) ^b^	4.0	3.2	
Types of infertility			
Primary infertility			111 (52.9)
Secondary infertility			99 (47.1)
Female baseline hormone level			
FSH (IU/l)	7.5	2.6	
LH (IU/l)	5.2	3.4	
Estradiol (pg/ml)	50.8	43.7	
Progesterone (ng/ml)	0.7	1.0	
AMH (ng/ml) ^c^	3.6	2.8	
AFC ^d^	19.7	11.3	
Ovarian stimulation protocol			
Long protocol			59 (28.1)
Prolonged protocol			85 (40.5)
Antagonist protocol			66 (31.4)
Treatment method			
IVF			154 (73.3)
ICSI			56 (26.7)
Sperm source			
Husband			153 (72.9)
Donor			57 (27.1)

^a^BMI was recorded in 208 participants. ^b^Duration of infertility was documented in 187 participants. ^c^AMH was detected in 189 participants. ^d^AFC was monitored only in 141 participants. AFC, antral follicle count; AMH, anti-Müllerian hormone; ICSI, intracytoplasmic sperm injection; IVF, *in vitro* fertilization; PPOS, progestin-primed ovarian stimulation; SD, standard deviation.

### Characteristics and biological properties of *circDDX10*

**Figure 1:**
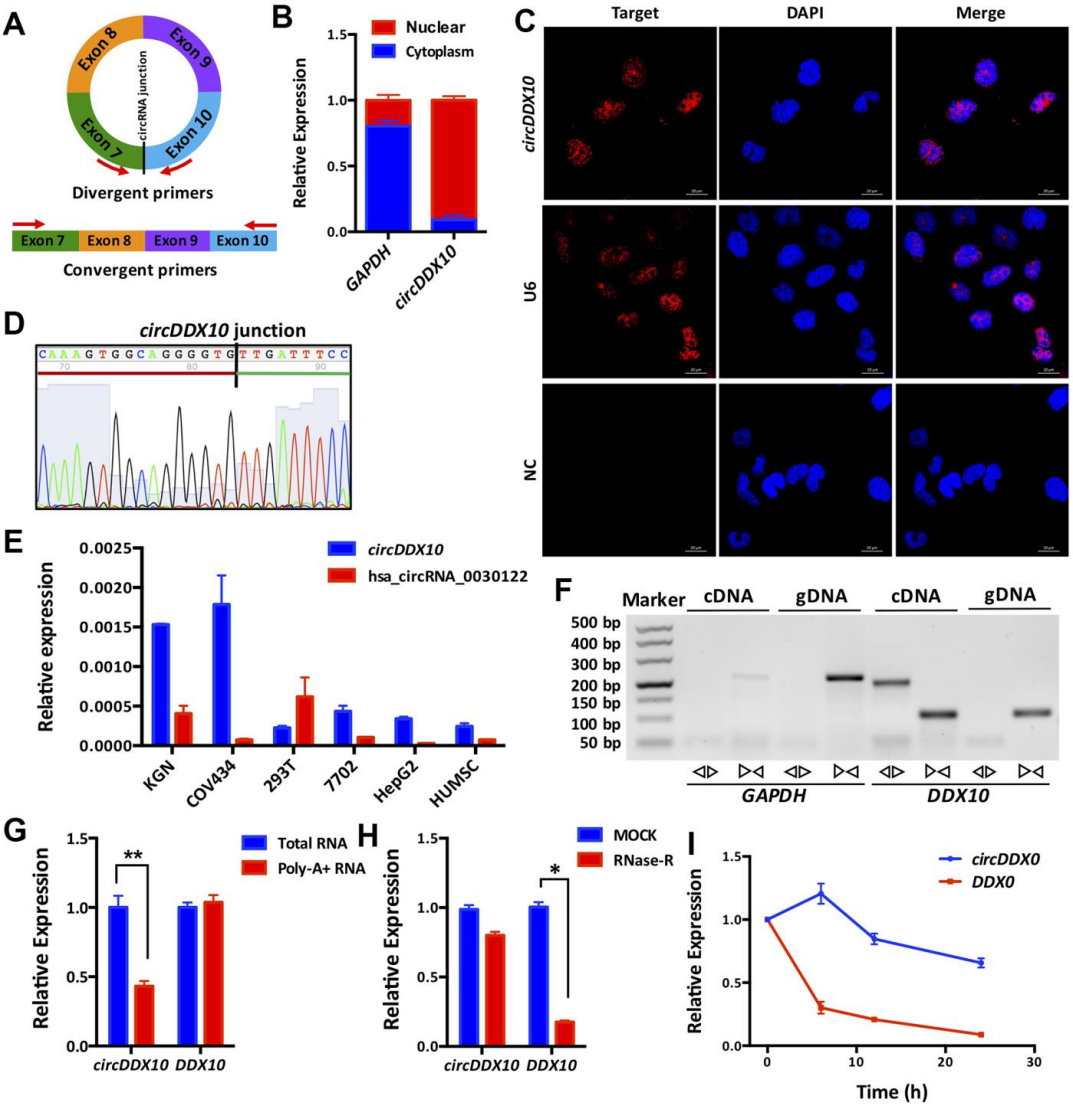
**Subcellular localization, expression levels and biological properties of *circDDX10* in granulosa cells (GCs).** (**A**) Schematics of back-to-back and face-to-face primers (black vertical line indicates the back-splicing site; red arrow represents the amplification direction of the upstream and downstream primers). (**B**) *CircDDX10* nucleoplasm distribution in COV434 (granulosa cell line). (**C**) Subcellular localization of *circDDX10* in COV434 using FISH technique. (U6 was used as a positive control for nuclear. NC, negative control. Bar = 20 μm). (**D**) Sanger sequencing of *circDDX10*. (Black vertical line indicates the back-splicing site. Red horizontal line representing 3' end and green horizontal line represents 5' end). (**E**) The relative expression levels of *circDDX10* in different cell lines (hsa_circRNA_0030122 was used as control). (**F**) Properties of back-splicing. (gDNA and cDNA were used as templates. And back-to-back primers and face-to-face primers were used for amplification. GAPDH as an internal control). (**G**) poly-A^-^ RNA characterization. (Total RNA and poly-A^+^ RNA were used for reverse transcription into cDNA, respectively). (**H**) Properties of resistance to RNase R digestion. (**I**) Properties of stability. All experiments were repeated for three times and the data were expressed as mean ± standard deviation. *, *P* < 0.05; **, *P* < 0.01.

Similar to the common circRNAs, *circDDX10* is back-spliced from “head” to “tail” of exon 7 and 10 of its host gene *DDX10* (Figure 1A, see detailed information of *circDDX10* in Supplementary Table 1). By isolating the nuclear and cytoplasmic RNA from GCs followed by quantitative RT–PCR (qRT–PCR), we found that *circDDX10* was mainly located in the nucleus (Figure 1B). This was further confirmed by FISH and Sanger sequencing (Figure 1C, 1D). As derived from the ovary tissue, *circDDX10* was highly enriched in GC lines (KGN and COV434) but not in other cell lines (293T, 7702, HepG2 and HUMSC), suggesting its tissue-specific expressing pattern (Figure 1E). We then validated the general biological characteristics of *circDDX10*. As the results demonstrated, *circDDX10* should be amplified *in vitro* by using back-to-back primers and cDNA as a template. However, it could not be amplified using face-to-face primers and/or gDNA as a template (Figure 1F). circRNA was successfully reverse-transcribed and amplified *in vitro* using total RNA, but could not be amplified after reverse transcription using Poly-A^+^ RNA (Figure 1G). Similar to other circRNAs, *circDDX10* was also resistant to RNase R digestion, whereas its corresponding linear transcript was rapidly degraded by RNase R (Figure 1H). The level of *circDDX10* was stable within 24 h at room temperature, while its corresponding linear transcript rapidly degraded (Figure 1I).

### Expression levels of *circDDX10* in GCs from human follicular fluids

**Figure 2:**
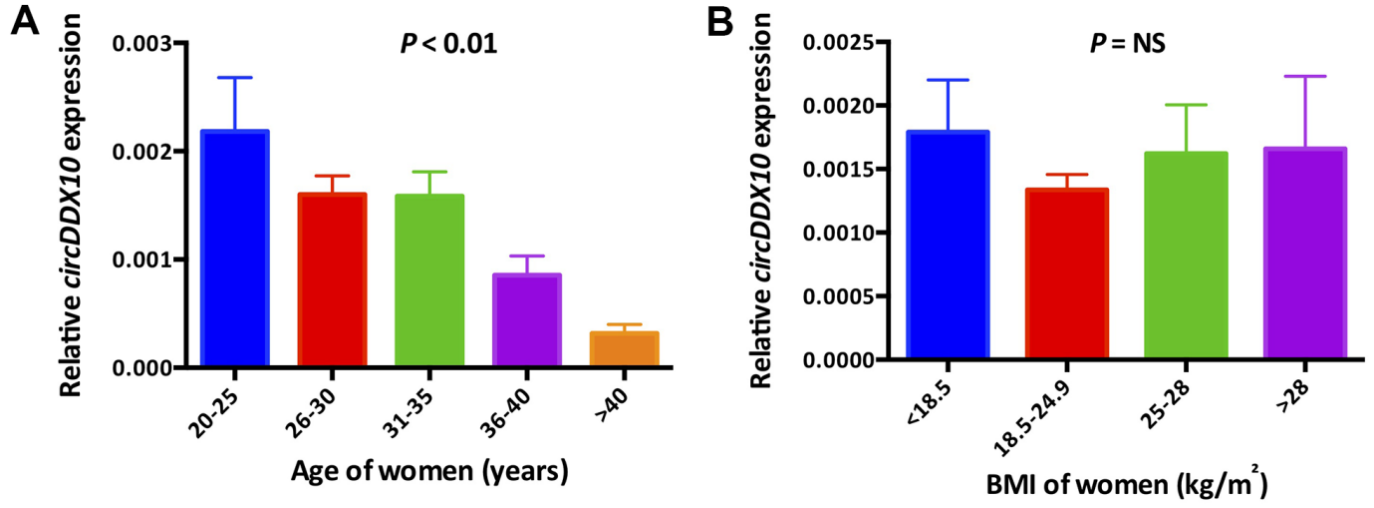
The expression levels of *circDDX10* in granulosa cells from 210 human follicular fluid stratified according to female age (**A**) and body mass index (**B**). BMI, body mass index.

**Figure 3:**
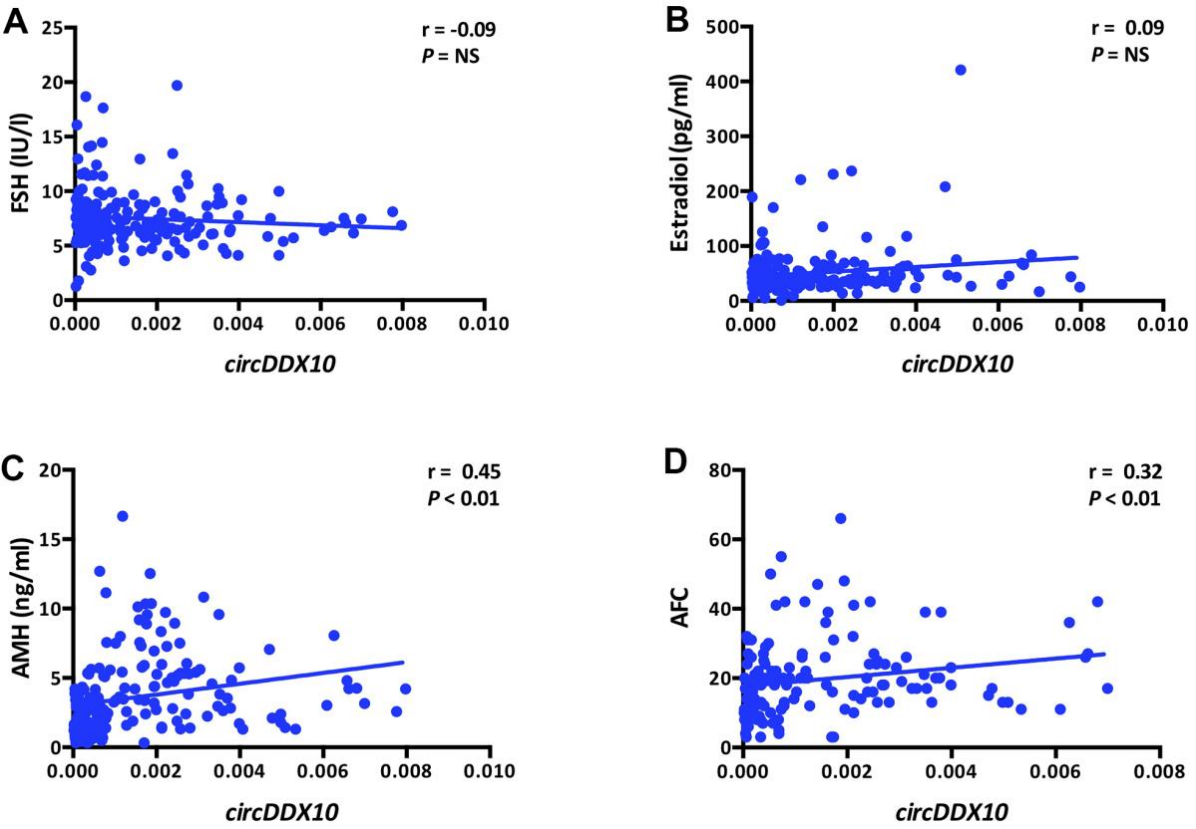
**Correlations between the levels of circDDX10 in granulosa cells (GCs) from human follicular fluid and reproductive hormones (FSH, estradiol and AMH, A–C) and antral follicle counts (AFC, D).** Serum FSH, LH, estradiol and progesterone levels were tested in all women (n = 210). AMH level and antral follicle count were assayed in 189 and 141 women, respectively.

The expression level of *circDDX10* in GCs from human follicular fluids decreased steadily with aging (*P* < 0.01, Figure 2A), whereas no significant differences were discovered with BMI (*P* > 0.05, Figure 2B). Further analysis demonstrated that there was no remarkable correlation between the level of *circDDX10* in GCs from human follicular fluids and FSH or estradiol (both *P* > 0.05, see in Figure 3A, 3B). However, it was positively correlated with AMH (r = 0.45, *P* < 0.01, Figure 3C) and antral follicle count (AFC) (r = 0.32, *P* < 0.01, Figure 3D).

### Association between *circDDX10* levels and ovarian reserve

A subgroup analysis of *circDDX10* expression levels of the 210 human follicular GC samples divided by the quartiles (Q1, Q2, Q3, Q4) showed significant differences among the age, AMH, AFC, and infertility years (*P* < 0.01). That is, the higher the age and the longer the infertility duration, the lower the expression level of *circDDX10*. Additionally, the higher the AMH levels and AFC, the higher the expression level of *circDDX10*. No significant differences were exhibited in the subgroups regarding BMI, FSH, and estradiol (*P* > 0.05, Table 2).

**Table 2:** Association between the levels of *circDDX10* in granulosa cells (GCs) from human follicular fluids and the baseline reproductive parameters.

**Parameters**	**Quartile of the levels of circDDX10 in GC from human follicular fluid**				***P* value**
	**Q1**	**Q2**	**Q3**	**Q4**	
	**n = 52**	**n = 53**	**n = 53**	**n = 52**	
Age (years)	32.9 (31.6–34.2)	33.7 (32.2–35.1)	30.2 (29.1–31.2)	29.9 (28.8–31.0)	< 0.01
BMI (kg/m^2^)	22.3 (21.3–23.3)	22.3 (21.3–23.3)	22.8 (21.9–23.7)	21.7 (20.6–22.8)	NS
FSH (IU/l)	7.8 (7.0–8.6)	7.9 (7.1–8.7)	7.0 (6.6–7.4)	7.4 (6.6–8.1)	NS
Estradiol (pg/ml)	44.7 (36.8–52.6)	47.8 (39.3–56.3)	49.6 (38.3–60.8)	61.6 (43.1–80.1)	NS
AMH (ng/ml)^a^	2.0 (1.7–2.4)	2.5 (1.8–3.1)	5.5 (4.5–6.6)	4.4 (3.7–5.1)	< 0.01
AFC^b^	14.9 (12.2–17.6)	16.9 (13.1–20.7)	25.4 (20.2–30.6)	21.4 (18.7–24.1)	< 0.01
Duration of infertility (years)^c^	4.6 (3.5–5.7)	5.0 (3.9–6.1)	3.4 (2.8–4.0)	3.1 (2.2–4.0)	< 0.01

^a^AMH was detected in 189 participants. ^b^AFC was monitored only in 141 participants. ^c^Duration of infertility was recorded in 187 participants. Data are shown as mean (95% CI). AFC, antral follicle count; AMH, anti-Müllerian hormone; GC, granulosa cell; NS, not statistically significant; Q, quartile.

### Correlation of *circDDX10* levels and assisted reproductive technology outcomes (ART)

Considering the ART outcomes, we found remarkable differences in the number of oocytes retrieved in each subgroup. The number of oocytes retrieved in Q3 and Q4 were significantly higher than those in the Q1 and Q2 subgroups (*P* < 0.01). In terms of normal fertilization rate, no significant difference was observed between the groups (*P* > 0.05), regardless of the overall fertilization rate, the IVF cycle, or the ICSI cycle, respectively. As for the good quality embryo rate, similar findings were observed. Specifically, the good quality embryo rate in the Q3 and Q4 groups were significantly higher than in the Q1 group (*P* < 0.05). Nevertheless, no significant differences were shown regarding the positive βhCG rate and clinical pregnancy rate, although they appeared to be positively correlated as a whole (*P* > 0.05, Table 3).

**Table 3:** Association between the levels of *circDDX10* in granulosa cells (GCs) from human follicular fluids and reproductive outcomes of assisted reproductive technology (ART).

**Parameters**	**Quartile of the level of circDDX10 in GC from human follicular fluid**								***P* value**
	**Q1**	**%**	**Q2**	**%**	**Q3**	**%**	**Q4**	**%**	
**Oocytes retrieved**									
Total	483	–	461	–	683	–	642	–	–
Mean	9.9 (7.8–11.9) ^a^	–	9.4 (7.7–11.1) ^a^	–	13.4 (11.7–15.) ^b^	–	12.6 (10.9–14.3) ^b^	–	< 0.01
**Normal fertilization rate**									
Total	266/452	58.8	284/451	63.0	404/640	63.1	367/597	61.5	NS
IVF cycles	142/268	53.0	209/337	62.0	301/496	60.7	298/505	59.0	NS
ICSI cycles	124/184	67.4	75/114	65.8	103/144	71.5	69/92	75.0	NS
**Good quality embryo rate**									
Total	126/266 ^a^	47.4	156/284 ^a, b^	54.9	259/404 ^b, c^	64.1	240/367 ^c^	65.4	< 0.01
IVF cycles	74/142 ^a^	52.1	124/209 ^a, b^	59.3	202/301 ^b^	67.1	196/298 ^b^	65.8	< 0.01
ICSI cycles	52/124 ^a^	41.9	32/75 ^a, b^	42.7	57/103 ^a, b^	55.3	44/69 ^b^	53.6	< 0.05
**β-hCG positive rate**									
Total	14/23	60.9	18/25	72.0	22/28	78.6	21/25	84.0	NS
IVF cycles	10/17	58.8	13/18	72.2	16/21	76.2	19/21	90.5	NS
ICSI cycles	4/6	66.7	5/7	71.4	6/7	85.7	2/4	50.0	NS
**Clinical pregnancy rate** ^*^									
Total	9/23	39.1	11/21	52.4	19/27	70.4	18/25	72.0	NS
IVF cycles	8/17	47.1	8/15	53.3	13/20	65.0	16/21	76.2	NS
ICSI cycles	1/6	16.7	3/6	50.0	6/7	85.7	2/4	50.0	NS

Mean oocytes retrieved are shown as mean (95% CI). a–c, denote the differences between groups (Kruskal-Wallis test and Bonferroni method were used for multiple comparisons, respectively). ^*^ A total of five patients’ clinical pregnancy outcomes were unclear. Normal fertilization rates were calculated as: number of 2PN/number of oocytes inseminated. Good quality embryo rates were calculated as: number of good quality embryos/number of 2PN embryos. GC, granulosa cell; ICSI, intracytoplasmic sperm injection; IVF, *in vitro* fertilization; NS, not statistically significant; Q, quartile; 2PN, two pronuclei.

### *circDDX10* modulated apoptosis and proliferation of GCs

**Figure 4:**
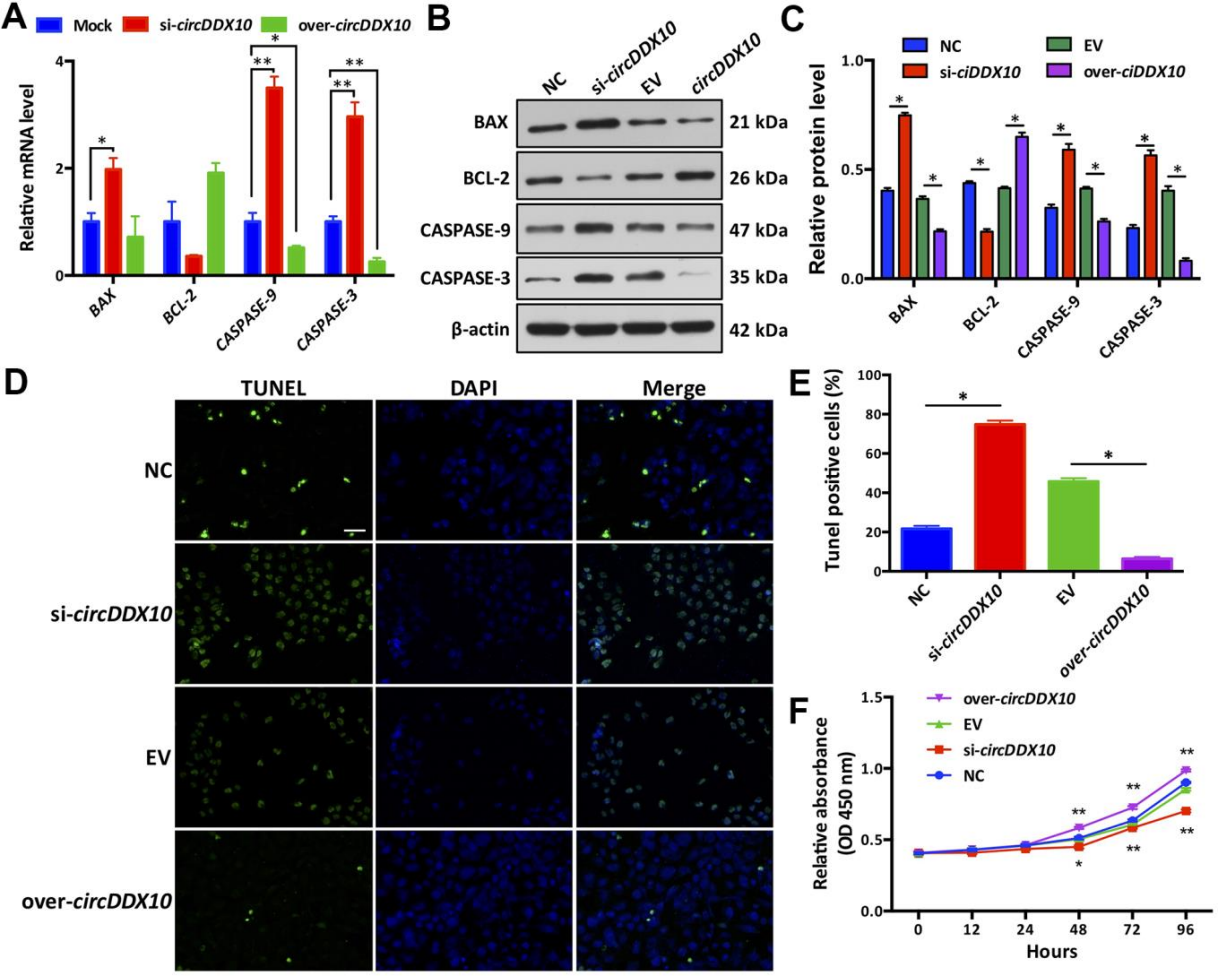
**Effect of *circDDX10* on apoptosis and viability of granulosa cells (GCs).** (**A**) Expression levels of apoptosis-related genes. Mock was a negative control group; (**B**, **C**) Expression level of apoptosis-related proteins (200×, bar = 100 μm). (**D**, **E**) Cell apoptosis was determined by TUNEL. (**F**) CCK-8 kit was used to detect the cell viability of each group *in vitro* for 24 h, 48 h, 72 h and 96 h, respectively. NC was a siRNA negative control and EV was an empty vector control group. Each set of experiments was repeated for three times. *, *P* < 0.05; **, *P* < 0.01.

Silencing *circDDX10* resulted in the up-regulation of apoptosis-related genes *BAX*, *CASPASE-3,* and *CASSASE-9* in GCs (*P* < 0.05), but it had no significant effect on the expression of *BCL-2* (*P* > 0.05). Overexpression of *circDDX10* significantly inhibited the levels of *CASPASE-3* and *CASSASE-9* (*P* < 0.05), while no remarkable changes were demonstrated on the levels of *BAX* and *BCL-2* (*P* > 0.05, Figure 4A). Similar changes on the translational level were confirmed. Low expression of *circDDX10* promoted the expression of apoptosis-related proteins BAX, CASSASE-3, and CASSASE-9 in GCs, while inhibiting the expression of the BCL-2 protein. Overexpression of *circDDX10* played an opposite role (Figure 4B, 4C). Results of TUNEL further supported the above findings (Figure 4D, 4E).

We then observed the effects of *circDDX10* on the proliferation activity of granulosa cells by using a CCK-8 kit. As the results demonstrated, no significant differences were exhibited on the proliferation activities among the four groups before 48 h of culturing *in vitro* (*P* > 0.05). However, this situation changed after 48 h of culturing. The proliferation activity of GCs in the si-*circDDX10* group significantly decreased, while a steady increase of proliferation activity of GCs in the over-*circDDX10* group was exhibited (*P* < 0.05, Figure 4F).

### *circDDX10* regulated steroidogenesis of GCs

**Figure 5:**
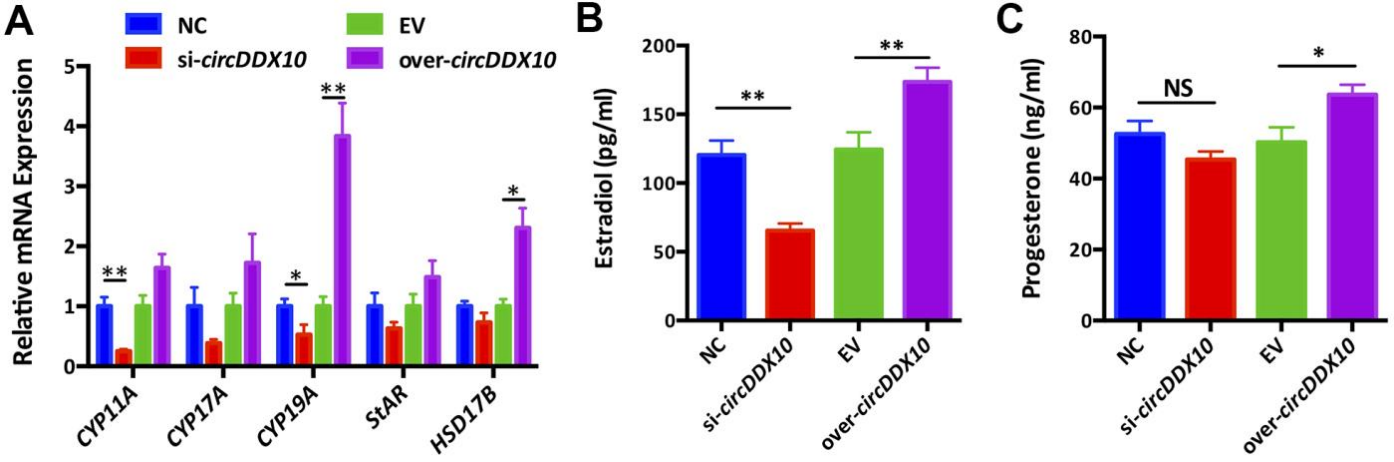
**The effect of *circDDX10* on steroid hormone synthesis of granulosa cells (GCs).** (**A**) The effect of *circDDX10* on the expression of steroid hormone synthesis related genes. The effect of *circDDX10* on the levels of estradiol (**B**) and progesterone (**C**). NC was a siRNA negative control and EV was an empty vector control. Each set of experiments was repeated for three times. *, *P* < 0.05; **, *P* < 0.01.

Silencing *circDDX10* significantly inhibited the expression levels of *CYP11A1* and *CYP19A1* (*P* < 0.05), but it did not change the levels of *CYP17A1*, *StAR,* and *HSD17B1* (*P* > 0.05). Overexpression of *circDDX10* promoted the levels of *CYP19A1* and *HSD17B1* (*P* < 0.05), but no significant changes were exhibited in the expressions of *CYP11A1*, *CYP17A1,* and *StAR* (*P* > 0.05, Figure 5A).

We further detected the changes of estradiol and progesterone levels in the supernatants obtained from each group using chemiluminescence immunoassay. The results demonstrated that the concentration of estradiol was significantly down-regulated in the si-*circDDX10* group (Figure 5B, 5C, *P* < 0.01). However, no significant effect on the concentration of progesterone was observed (*P* > 0.05). Conversely, overexpression of *circDDX10* significantly up-regulated the estradiol (*P* < 0.01) and progesterone levels (*P* < 0.05).

## DISCUSSION

Age has been a major factor affecting clinical ART outcomes [19]. This study found that the expression level of *circDDX10* in human GCs from follicular fluid was negatively correlated with age, but not with BMI. This is consistent with the results of the RNA sequencing in our previous work [18]. That is, *circDDX10* shows a down-regulation trend in the aged ovarian tissue, which suggests that *circDDX10* may be one of the factors involved in the regulation of ovarian function.

AMH is expressed in GCs of pre-antral follicles and antral follicles. Compared with FSH, AMH is not affected by menstrual cycle, and its expression is more stable [20]. AMH released from GCs of antral follicles is measurable in serum, and its concentration has been shown to be proportional to the number of follicles developed in the ovary [21]. Currently, AMH is recognized as the gold standard for ovarian reserve function and predicting ovarian response to stimulating drugs [20, 22]. Evidences have shown that antral follicle count (AFC) is associated with female reproductive age in fertile women [23]. This study found that the expression level of circDDX10 in GCs derived from human follicular fluid has a positive correlation with serum AMH levels and AFC in infertile women. This suggests that it may be closely related to ovarian reserve function. However, the linear relationship seemed not so strong. To some extent, this could be due to the biological differences between individuals. Moreover, some of the patients presented with polycystic ovarian morphology (PCOM) in response to ovarian stimulation. Lastly, the limited cases included in this study also made our findings not strong enough. At present, the specific relationship between *circDDX10* and AMH expressed in GCs is still unclear, and whether there is an interaction between them needs further investigation.

Recently, Cheng et al. [15] discovered and identified two circRNAs (circRNA_103827 and circRNA_104816) in human GCs from follicular fluid that may be associated with reproductive aging. Bioinformatics analysis revealed these two circRNAs were closely related to glucose metabolism. The mitotic cycle and ovarian steroid hormone synthesis may be potential indicators of an impaired follicular microenvironment. Among them, the expression level of circRNA_103827 was significantly up-regulated with age. It was also closely related to the good quality embryo rate and live birth rate in IVF cycles, but its specific mechanism of action has not yet been elucidated. However, by comparing the sequencing results of this study, we did not find the two circRNAs identified by Cheng et al., which may be related to the specimens used in this study and the methods adopted for sequencing. In this study, ovarian cortex specimens were used for sequencing, whereas Cheng et al. used GC specimens derived from follicular fluids. In our study, we used circRNA high-throughput sequencing methods, while Cheng et al. used gene chip technology. There might also be differences in the methods of bioinformatics analysis, prediction, and screening. Our results showed that the expression level of *circDDX10* in human follicular GCs was correlated with the number of eggs obtained and the good quality embryo rates. In the high expression levels of the *circDDX10* groups (Q3 and Q4), the number of eggs obtained and good quality embryo rates were higher than those in lower level groups (Q1 and Q2). In terms of pregnancy outcomes, βhCG positive rate and clinical pregnancy rate were not statistically different. This may be related to the lack of follow-up period and the limited number of cases included in this study. Therefore, more studies are still needed to clarify the correlation between the levels of *circDDX10* and the clinical pregnancy outcomes of ART.

Proliferation and differentiation of GCs is an important part of follicular development [24]. After the primordial follicles are activated, the GCs then express FSHR. FSHR-conjugated G protein on GCs stimulates hormone-sensitive adenylate cyclase after stimulation with FSH. This results in increased intracellular cAMP levels, which further activate the downstream PKA signaling pathway [25, 26]. Apoptosis of GCs is closely related to follicular atresia and it is critical for the selection of dominant follicles, affecting the function of normal ovarian function [3, 27]. *BAX* and *BCL-2* are important genes that regulate GC apoptosis. The BCL-2 protein has anti-apoptotic functions, while the BAX protein can accelerate the induction of GC apoptosis. The dynamic balance is very important for the normal development of follicles [28, 29]. The mechanism of apoptosis is tightly regulated by members of the BCL-2 protein family. In general, the BAX protein forms a heterodimer with BCL-2 to exert biological effects, and the ratio of intracellular BCL-2/BAX can be used as a predictor of cell fate. When the concentration of the BCL-2 protein in the cells is increased, the proportion of BCL-2/BAX is also increased, and the amount of the BCL-2/BAX heterodimer is increased, as well. This can inhibit the occurrence of apoptosis. Conversely, when the proportion of BCL-2/BAX decreases and intracellular mitochondrial membrane permeability increases, the process of apoptosis is accelerated [30, 31]. The CASPASE family consists of important apoptotic proteins, and the most important apoptosis effector protein is CASPASE-3, which can participate in various pathways to induce apoptosis. When intracellular death signalling occurs, it interacts with BCL-2 family members such as BAX, BAK, etc. This activates BCL-2, causing increased mitochondrial membrane permeability, decreased mitochondrial membrane potential, and an increased release of cytochrome C and other proteins. Pigment C forms a multimeric complex by binding to an apoptotic protein activating factor (APAF-1). This recruits the CASPASE-9 precursor to form apoptotic bodies, as well as initiates the CASSASE cascade to activate downstream CASSASE-3 and CASSASE-6. CASSASE-7 (the ultimate performer of apoptosis) ultimately triggers the occurrence of apoptosis [32, 33]. Several studies have confirmed the important role of the BCL-2 family in ovarian apoptosis. The number of follicles in BCL-2 deficient mice is reduced [34], while overexpression of BCL-2 leads to decreased follicular cell apoptosis and atresia [35]. In BAX-knockdown mice, the number of abnormal follicles increased and the number of GCs contained was too large [36]. The BAX protein was significantly expressed in the atretic follicles compared with healthy ovarian follicles [37]. In mice and pigs, CASPASE-9 and APAF-1 have been shown to cause follicular atresia [38, 39]. Luo et al. [40] found that GC activity significantly decreased after heat stress (43° C). Additionally, apoptosis-related gene CASPASE-3 mRNA and protein levels were found in a model of ovarian injury induced by heat stress in mice. The expression level significantly increased. The BAX/BCL-2 ratio also significantly increased. The results of this study demonstrated that the silencing of circDDX10 resulted in increased expression of pro-apoptotic factors BAX, CASPASE-3, and CASSASE-9 in GCs, as well as decreased expression of apoptotic factor BCL-2. Meanwhile, overexpression of *circDDX10* significantly inhibited BAX and CASSASE- 3. The expression of CASSASE-9 increased the expression of BCL-2. The TUNEL results also support the above findings. This suggests that with the progression of ovarian aging, the decreased levels of *circDDX10* can lead to the increased expression of apoptosis proteins BAX, CASSASE-3, and CASSASE-9, as well as decreased levels of anti-apoptotic protein BCL-2, thus inducing GC apoptosis. The increased apoptosis of GC in turn affects the development of oocytes, which may be one of the mechanisms by which *circDDX10* regulates GC apoptosis and participates in ovarian aging.

Hormones can regulate the metabolic activities of various cells and affect the metabolism, growth, and development of organisms. Luteinizing hormone secreted by the pituitary gland during the mid-menstrual period accelerates the production of estradiol and progesterone in GCs, which are essential for uterine receptivity and embryo implantation [41, 42]. In the process of progressive occlusion of follicles, steroid hormones can also be involved in the apoptotic process of GCs as an important influencing factor. As one of the important steroid hormones, estradiol can significantly inhibit the apoptosis of antral follicles and early antral follicles [43]. Progesterone regulates GC apoptosis through a G-like protease pathway [44]. The GSK-3β and ERK1/2 signaling pathways play an important role in the regulation of granule/luteocyte steroid hormone synthesis [45, 46]. To explore the mechanism by which *circDDX10* regulates the synthesis and secretion of steroid hormones, we examined the expression of five steroid hormone synthesis-related genes (*CYP11A*, *CYP17A*, *CYP19A*, *StAR*, and *HSD17B*). It is well known that StAR plays an important role in the transfer of cholesterol from the mitochondrial outer membrane to the mitochondrial inner membrane, thereby providing a substrate for the biosynthesis of steroid hormones. This is then converted to pregnenolone by the CYP11A enzyme [47, 48]. The conversion of androgen to estradiol requires the involvement of CYP19A [49]. HSD17B contributes to the conversion from estrone to estradiol [50]. It was reported that chemokine treatment can increase the phosphorylation of P38 MAPK in rat GCs, thereby up-regulating the transcription levels of StAR, CYP11A, and 3β-HSD [51]. In the Hsd17b1 knockout mouse model, researchers found that the ovarian Hsd17b enzyme activity was significantly reduced, suggesting a core role of Hsd17b1 in ovarian physiology. The loss of Hsd17b activity leads to an increase in the proportion of ovarian estrone/estradiol, a significant decrease in progesterone concentration in the ovary, and a significant change in the expression of StAR, Cyp11a1, Lhcgr, Hsd17b7, and Cyp17a1 [51]. Our study found that the silencing of *circDDX10* induces downregulation of *CYP11A*1 and *CYP19A1* genes involved in granulocyte steroid synthesis, whereas overexpression of *circDDX10* promotes expression of steroid hormone synthesis-related genes *CYP19A1* and *HSD17B1*. This in turn affects steroid hormone synthesis. Our results show that *circDDX10* may affect the quality and developmental potential of oocytes by affecting steroid synthesis, which may be one of the mechanisms involved in the regulation of ovarian aging.

### Advantages and limitations

In this study, the expression level of *circDDX10* in the GCs from follicular fluid was detected. Its correlation with ovarian reserve function and clinical outcomes of ART was analyzed. The expression level of *circDDX10,* ovarian reserve function, and ART were obtained for the first time in GCs from follicular fluid. There is a close relationship between the number of eggs and good quality embryo rates. Additionally, *circDDX10* may participate in the regulation of ovarian aging by regulating the proliferation and apoptosis of GCs, as well as the synthesis of steroid hormones. This provides a molecular mechanism for exploring non-invasive biomarkers of assisted reproduction and exploring ovarian aging. However, there are still some limitations in this study. First, the clinical follicular fluid specimens included are limited. Also, there are certain biological differences between individuals, which may interfere with the results. In addition, the follow-up period was not long enough for an analysis of clinical outcomes. Therefore, in the future, it is necessary to further extend the follow-up time by expanding the sample size and analyzing the correlation between the expression levels of *circDDX10* and ART clinical outcomes (including abortion rate, live birth rate, *etc*.). Furthermore, we used the cell line COV434 instead of human primary GCs for *in vitro* studies. COV434 could not completely mimic the biological function of human primary GCs [52]. Therefore, the interpretations of these results should be taken with caution. Finally, this study only focused on the role of *circDDX10* in the biological function of ovarian GCs. We have not observed the effect of *circDDX10* on the developmental potential of oocytes. Therefore, more experiments are needed to further explore the interacting proteins or genes of *circDDX10* in GCs. It is better for us to observe the effects of *circDDX10* on oocyte development by constructing a technique such as the *circDDX10* knockout model. Then, we can further clarify the specific molecular mechanism that *circDDX10* participates in with ovarian aging.

## CONCLUSIONS

In conclusion, we first identified *circDDX10*, an ovary-derived circRNA, which was stably expressed in GCs from human follicular fluids. Being closely related to female age, ovarian reserve, oocytes retrieved, as well as good quality embryo rates of women undergoing ART treatment, *circDDX10* could be a novel and valuable biomarker for predicting ART outcomes. By modulating the apoptosis/proliferation activity and steroid hormone biosynthesis, *circDDX10* might participate in the process of ovarian aging. However, in order to further uncover the mechanisms of ovarian aging, more studies on molecular levels are still needed to further elucidate the roles of *circDDX10* in ovarian aging.

## MATERIALS AND METHODS

### Sample collection and preparation

We collected follicular fluid samples from women undergoing oocyte retrieval for IVF/ICSI between February 2017 and March 2018 at the Center of Reproductive Medicine, Tongji Medical College, Huazhong University of Science and Technology. Participants were required to meet the following requirements: 1) age between 20 to 45 years old; 2) unable to conceive naturally for at least 1 year, regardless of male, female, both, or uncertain factors; 3) primary or secondary infertility; and 4) IVF or ICSI cycles fertilized by husband or donor sperm. The mean age of the participants was 35.4 ± 9.3 years old (range from 20 to 45, n = 239). Participants with polycystic ovary syndrome, premature ovarian failure, endometriosis, and other reproductive endocrine diseases, such as thyroid disease, diabetes, adrenal disease, *etc.*, were excluded. The basic information of the patients (n = 210) who qualified for the final analysis is presented in Table 1. This study was approved by the ethics committee of the Tongji Medical College, Huazhong University of Science and Technology (NO. 2016 (04) on April 28, 2016, Wuhan, China. Written informed consent was obtained from each participant in this study.

### Isolation, purification and identification of GCs

On the day of oocyte retrieval, all follicular fluid samples from the same patient were pooled after cumulus–oocyte complexes were isolated for conventional *in vitro* fertilization (IVF) or intracytoplasmic sperm injection (ICSI) procedures. GCs were individually isolated from follicular fluid samples and the purity of the GCs was confirmed using similar methods as previously described [18]. Since they are highly specialized primary luteinized granulosa cells, which produces large amounts of progesterone and estradiol hormones, they could not proliferate neither spontaneously nor after stimulation with a mitogenic agent [53]. The harvested GCs were stored in TRIzol reagent (Life Technologies, CA, USA) at -80° C until RNA extraction.

### Cell lines

The GC cell lines (KGN and COV434) were cultured, passaged, and cryopreserved according to previously described methods [54]. FSRH was used as a marker for identification of the GC cell line (Supplementary Figure 1). In this study, COV434 was obtained from a human granulosa cell tumor with specific biological characteristics, including the synthesis of 17 beta-estradiol in response to FSH, the absence of the LH receptor and luteinization capability, and the presence of specific molecular markers of apoptosis enabling the induction of follicular atresia [55]. The cultured COV434 cells were further divided into four groups according to the experimental design.

### Plasmid constructs and transient transfections

The siRNA specific interference sequence (si-*circDDX10*) was designed and synthesized according to the reverse splicing site of *circDDX10* (Suzhou GenePharma Co., Ltd, Suzhou, China). The overexpression vector of *circDDX10* (over-*circDDX10*) was designed and constructed by Guangzhou Geneseed Biology Co., Ltd. The GCs (COV434 cell line) were transient transfected using Lipofectamin 2000 (Invitrogen, USA). The specificity of siRNA and the overexpression vector were confirmed by qRT-PCR (see in Supplementary Figures 2, 3). A specific interfering sequence of the linear transcript was also designed to exclude the influence of linear transcription on the experimental results. The ineffective interfering sequence was used as a negative control. All of the information from the oligo sequences are shown in Supplementary Table 2.

### cDNA synthesis, PCR, electrophoresis and quantitative real-time PCR (qRT-PCR)

Total RNA was reverse transcribed using random primers with the PrimeScript RT reagent kit (RR047A, Takara, Dalian, China) following the manufacturer’s protocol. At least three pairs of divergent primers encompassing circRNA-specific back-splice junctions were designed for each of the candidate circRNAs. Details of the primer sequences are summarized in Supplementary Table 3. Only primers achieving a single peak in the melting curve were considered sufficient for the qRT-PCR validation. The qRT-PCR and validation experiments were performed using the methods as previously reported [18, 56].

### Genomic DNA extraction

Genomic DNA (gDNA) of the ovary samples was extracted using the TIANamp Genomic DNA Kit (DP304, TIANGEN BIOTECH, Beijing, China) following the manufacturer’s instructions. The gDNA fraction was immediately transferred to a 2 ml microfuge tube and stored until further processing at –80° C.

### RNase R treatment

Total RNA was incubated with RNase R (1 IU enzyme per μg RNA) in 1× RNase R buffer for 15 min at 37° C, and then quickly placed on ice. The control group was treated with an equal volume of ddH_2_O. DNase digestion, cDNA synthesis, and qRT-PCR procedures were performed as described above. qRT-PCR Ct values were calculated automatically and ∆Ct was termed as Ct (RNase R treatment) − Ct (mock treatment). The expression of circRNAs and mRNAs before the RNase R treatment were normalized as 1. 2^−∆Ct^ was used to compare the expression of circRNAs and mRNAs after the RNase R digestion.

### PolyA^+^ RNA extraction

PolyA^+^ RNA was enriched using the kit (Magnosphere^TM^ UltraPure mRNA Purification Kit, Takara, Dalin, China), according to the manufacturer’s protocol manual. The polyA^+^ RNA fraction was immediately transferred to a 2 ml microfuge tube and stored at –80° C until the next step.

### Detection of cellular proliferation and apoptosis

A CCK-8 cell proliferation detection kit ((DOJINDO, Shanghai)) was used to detect the proliferation activity of GCs after transient transfection at 0 h, 24 h, 48 h, 72 h and 96 h, respectively, following the manufacturer’s instructions. The GCs were formalin-fixed 72 h after transfection. The TUNEL method was used to detect the apoptosis of COV434 cells, according to the manufacturer's instructions (Roche, Switzerland).

### Chemiluminescence immunoassay

The supernatant of the GCs was collected after centrifugation for 15min at 3500 rpm. Next, we quantified the levels of estradiol and progesterone in the supernatant by using Chemiluminescence Quantitative Immunoassay Kits (Beckman Coulter, USA), and we did so following the manufacturer's instructions as previously described [57].

### Western blotting

The cultured GCs were lysed using RIPA protein extraction reagent (Beyotime, Beijing, China) supplemented with a protease inhibitor cocktail (Roche, CA, USA). Protein quantification was determined by the Bradford method (Beyotime, Shanghai, China). Approximately 40 μg of protein extract was separated by 10% SDS-polyacrylamide gel electrophoresis (SDS-PAGE) and then transferred to a nitrocellulose membrane (Sigma-Aldrich, MO, USA). After blocking for 2 h, membranes were incubated with primary antibody (1:1000; GAPDH, #GB12002, Servicebio, China; Caspase-3, #9662, CST, USA; Caspase-9, #GB11053-1, Servicebio, China; Bax, #GB11007, Servicebio, China; Bcl-2, #ab59348, Abcam, USA) overnight at 4° C. Membranes were washed and incubated for 1.5 h with HRP conjugated secondary antibodies (1:2000; ab6721, Abcam, Cambridge, MA, USA). ECL chromogenic substrate was used to visualize the bands, and the band intensity was measured using Image J (version 1.49s, National Institutes of Health, USA).

### Statistical analysis

Statistical analyses were performed using SPSS Statistics (version 23.0; IBM, Armonk, NY, USA) and GraphPad Prism 6.0 (version 6.0c; GraphPad Software, Inc., San Diego, CA, USA). All of the data are displayed as the mean ± SEM for triplicate independent measurements. One-way ANOVA was used to assess the differences between groups. Differences with *P* values < 0.05 were considered statistically significant.

## Supplementary Material

Supplementary Figures

Supplementary Tables
