# Supplementary material for: Circular DDX10 is associated with ovarian function and assisted reproductive technology outcomes through modulating the proliferation and steroidogenesis of granulosa cells
Source: Aging (Albany NY). 2021 Mar 19;13(7):9592–612. doi: 10.18632/aging.202699 (PMC8064152; doi:10.18632/aging.202699)
Supplement: Supplementary Figures [file aging-13-202699-s001.pdf]

SUPPLEMENTARY FIGURES

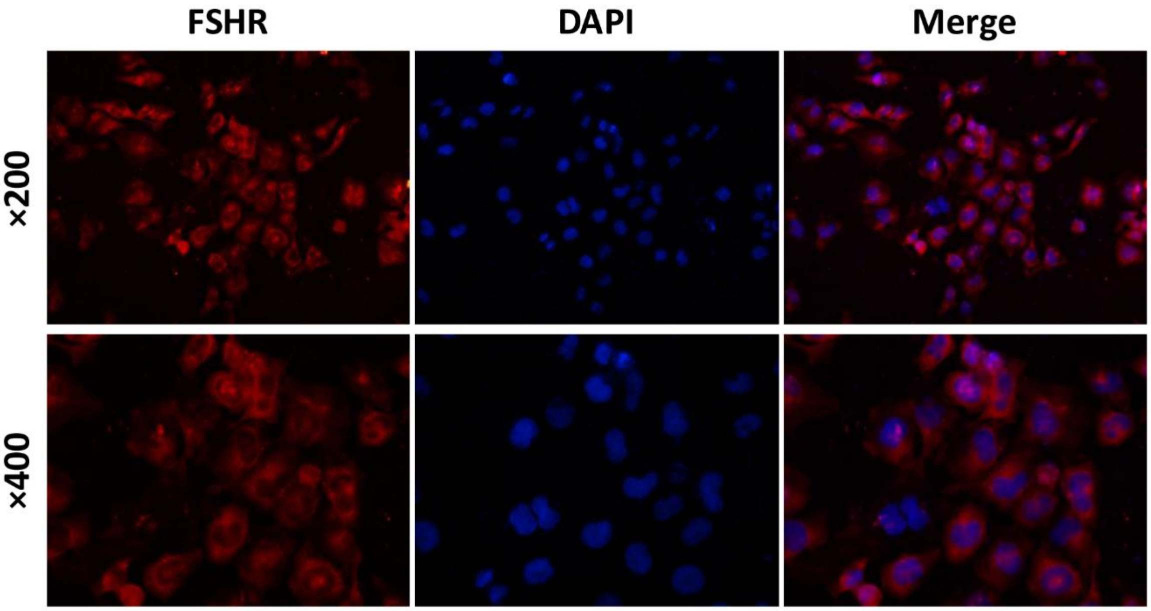

Supplementary Figure 1. Immunofluorescence of the COV434 cell line. FSHR was used as a biomarker for the COV434 cell line.

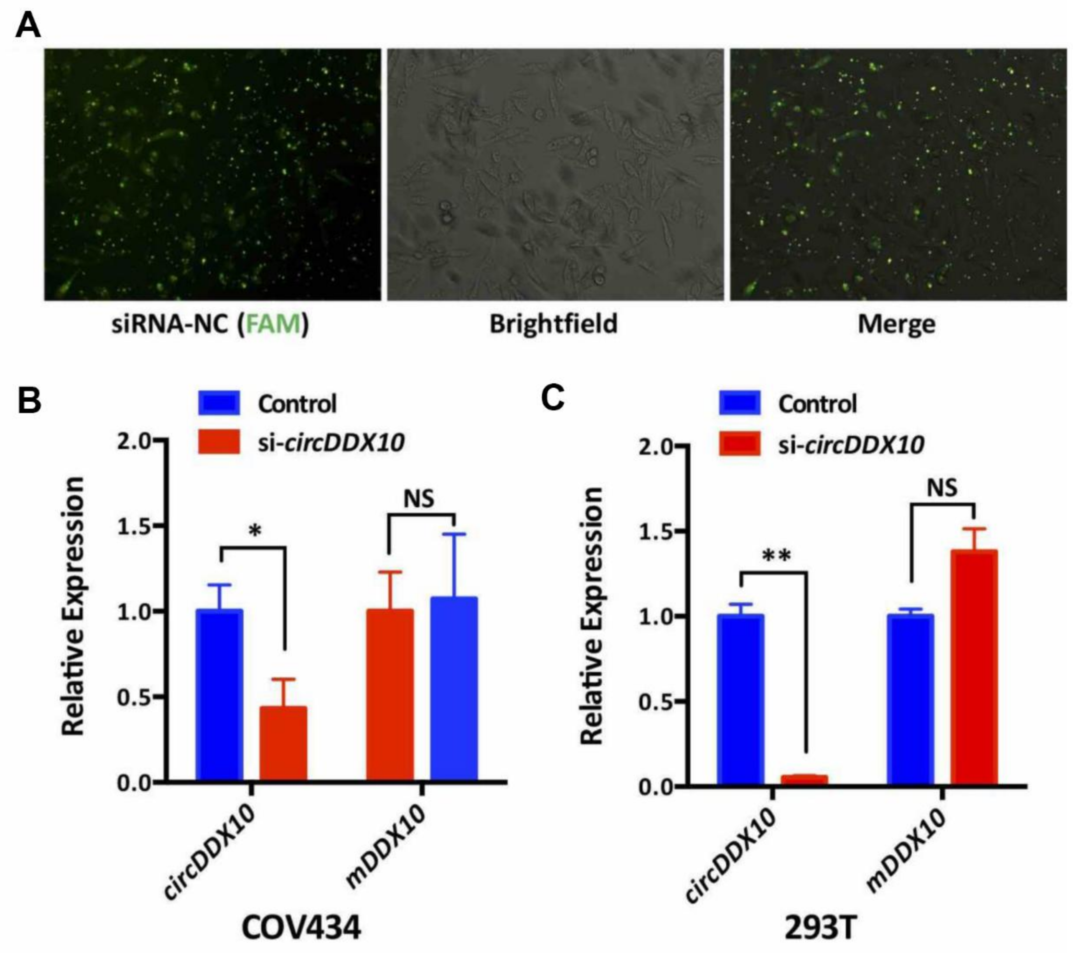

Supplementary Figure 2. si-circDDX10 specifically interferes with the expression of circDDX10. (**A**) Four to six hours after transfection with COV434 cell line using Lipofectamine 2000 reagents. siRNA-NC (FAM) with green fluorescence was used as negative control to evaluate the transfection effect. Brightfield, for bright field figure; Merge, for merged image. (**B**, **C**) indicate the relative expression levels of the circDDX10 in COV434 and 293T cells after transfection, respectively. GAPDH was used as an internal control. The null interference sequence was used as a negative control. And mDDX10 was used as a positive control. Each set of experiments was repeated for three times. *, P < 0.05; **, P < 0.01; NS, no statistical significance.

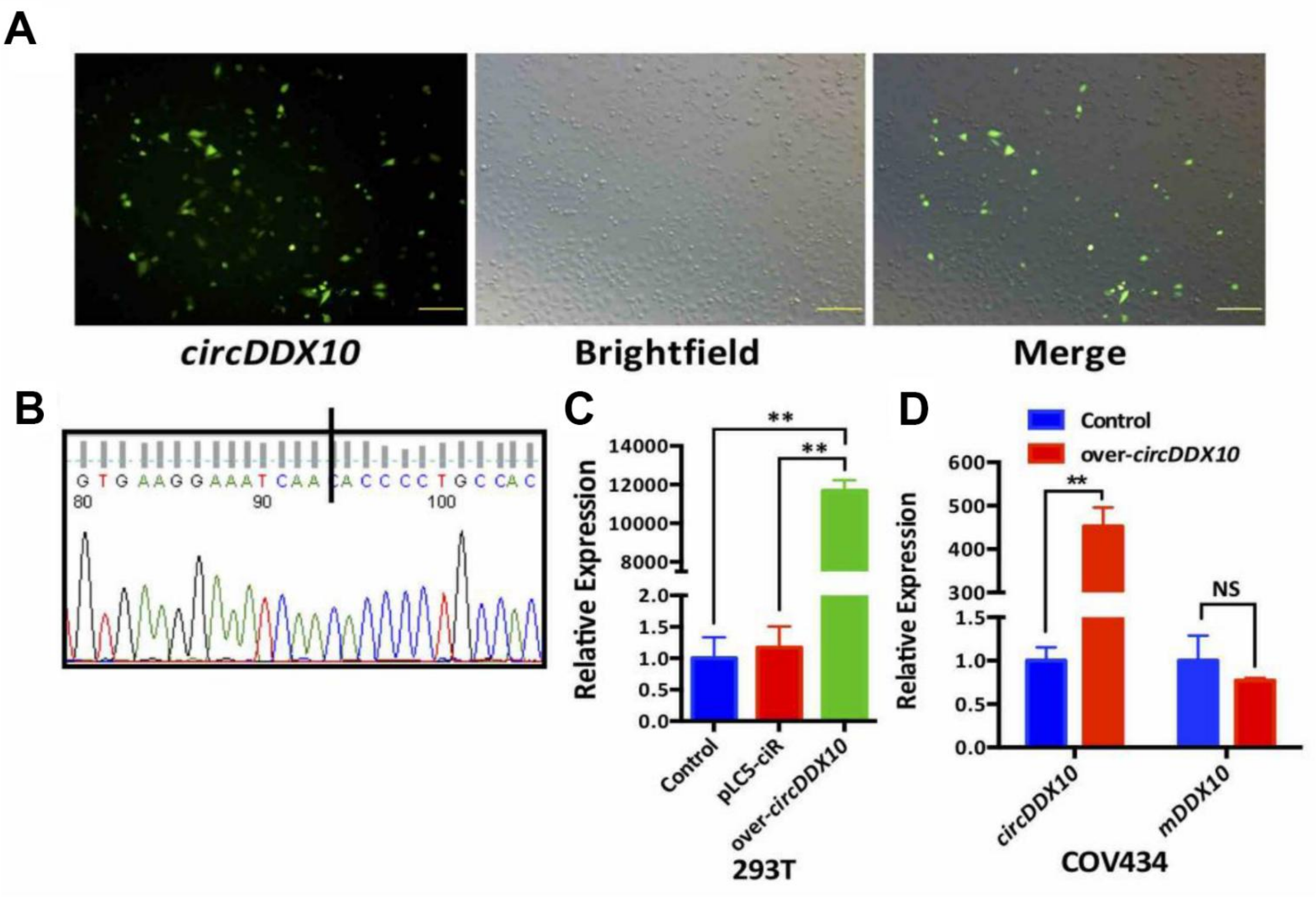

Supplementary Figure 3. Construction of the circDDX10 overexpression vector. (**A**) Lipofectamine 2000 transfected COV434 cell line after 24 h, the expression of green fluorescent protein pLC5-ciR detection transfection effect. Brightfield for bright field map, Merge for combined images. (**B**) Sanger sequence of circDDX20. (**C**) The relative expression of circDDX10 after transfection with over-circDDX10 vector into 293T cell line. GAPDH was used as an internal control, Control was a completely blank control group, and plasmid empty vector was used as a negative control. (**D**) The relative expression of circDDX10 after transfection with over-circDDX10 vector into COV434 cell line. GAPDH as an internal control, Control as a completely blank control group, mDDX10 as a positive control. Each set of experiments was repeated for three times. *, $P < 0.05$; **, $P < 0.01$; NS, no statistical significance.
