# Supplementary material for: Circular DDX10 is associated with ovarian function and assisted reproductive technology outcomes through modulating the proliferation and steroidogenesis of granulosa cells
Source: Aging (Albany NY). 2021 Mar 19;13(7):9592–612. doi: 10.18632/aging.202699 (PMC8064152; doi:10.18632/aging.202699)
Supplement: Supplementary Tables [file aging-13-202699-s002.pdf]

## SUPPLEMENTARY TABLES

**Supplementary Table 1. Detail information of *circDDX10* in the gene bank.**

| Name | *circDDX10* |
| --- | --- |
| Transcript ID | hsa_circRNA_0024246 |
| Position | chr11:108559663-108577564 (+) |
| Best transcript | NM_004398 |
| Gene Symbol | DEAD-box helicase 10 (*DDX10*) |
| Exon Composition | 7–10 |
| Genomic Length (nt) | 17902 |
| Spliced Length (nt) | 474 |
| Primer Sequence | F: 5'-GGAAATCAACACCCCTGCCA-3'<br>R: 5'-CGACCATGGAGTGCAAGGAT-3 |
| AT (° C) | 60 |
| Product Length (bp) | 204 |

AT: annealing temperature.

**Supplementary Table 2. siRNA and overexpressed vector sequences used in this study.**

| Oligo | Sequences |
| --- | --- |
| *siRNA-circDDX10-1* | Sense: AAAUCAACACCCCUGCCACTT<br>Antisense: GUGGCAGGGGUGUUGAUUUTT |
| *siRNA-circDDX10-2* | Sense: AGGAAAUCAACACCCCUGCTT<br>Antisense: GCAGGGGUGUUGAUUUCCUTT |
| *siRNA-*m*DDX10* | Sense: GCUGCAGUACUCUUUGCUATT<br>Antisense: UAGCAAAGAGUACUGCAGCTT |
| *siRNA-negative control (FAM)* | Sense: UUCUCCGAACGUGUCACGUTT<br>Antisense: ACGUGACACGUUCGGAGAATT |
| *siRNA-negative control* | Sense: UUCUCCGAACGUGUCACGUTT<br>Antisense: ACGUGACACGUUCGGAGAATT |
| *siRNA-positive control (GAPDH)* | Sense: UGACCUCAACUACAUGGUUTT<br>Antisense: AACCAUGUAGUUGAGGUCATT |
| overexpressed-*circDDX10* | F: cgGAATTCTAATACTTTCAGCACCCCTGCCACTTTGGAACAGA<br>R: cgGGATCCAGTTGTTCTTACTTGATTTCCTTCACAGGTACTT |

**Supplementary Table 3. Primer sequences for qRT-PCR in this section.**

| Gene | Gene bank access number | Primer sequences | Product length (*bp*) | AT (° C) |
|---|---|---|---|---|
| *GAPDH* | / | F: GAAGGTGAAGGTCGGAGTC<br>R: GAAGATGGTGATGGGATTTC | 226 | 60 |
| *CYP11A1* | NM_000781.2 | F: CAGTCATCCTAGCAGTCCCC<br>R: GGGGATCTCATTGAAGGGGC | 216 | 60 |
| *CYP17A1* | NM_000102.3 | F: TGAGTTTGCTGTGGACAAGG<br>R: GGATTCAAGAAACGCTCAGG | 114 | 60 |
| *CYP19A1* | DQ118405.1 | F: ATGAAAGCTCTGTCAGGCCC<br>R: TCAACACGTCCACATAGCCC | 121 | 60 |
| *HSD17B1* | NM_000413.3 | F: ATGACGTTTATTGCGCCAGC<br>R: GGTGTTGACTCACTGGACCC | 95 | 60 |
| *StAR* | / | F: GGGATGAGGCTCTTGGATT<br>R: CCCATATCAGCCACTAGCAT | 150 | 60 |
| *BCL-2* | / | F: GTGGAGGAGCTCTTCAGGGA<br>R: AGGCACCCAGGGTGATGCAA | 304 | 60 |
| *BAX* | / | F: CAGGATGCGTCCACCAAGAA<br>R: GCTCCCGGAGGAAGTCCAAT | 286 | 60 |
| *CASP3* | NM_004346 | F: CATGGAAGCGAATCAATGGACT<br>R: CTGTACCAGACCGAGATGTCA | 139 | 60 |
| *CASP9* | NM_032996 | F: CTCAGACCAGAGATTCGCAAAC<br>R: GCATTTCCCCTCAAACTCTCAA | 116 | 60 |

AT: annealing temperature. *, divergent primers.
